# Stress, Anxiety, and Change in Alcohol Use During the COVID-19 Pandemic: Findings Among Adult Twin Pairs

**DOI:** 10.3389/fpsyt.2020.571084

**Published:** 2020-09-25

**Authors:** Ally R. Avery, Siny Tsang, Edmund Y. W. Seto, Glen E. Duncan

**Affiliations:** ^1^Department of Nutrition and Exercise Physiology, Washington State University, Spokane, WA, United States; ^2^Department of Environmental and Occupational Health Sciences, University of Washington, Seattle, WA, United States

**Keywords:** novel coronavirus, alcohol use, perceived stress, anxiety, social restriction, twins

## Abstract

The novel coronavirus (COVID-19) has impacted the lives of people worldwide since being declared a pandemic on March 11, 2020. Social restrictions aimed at flattening the curve may be associated with an increase in stress and anxiety, which may increase the use of alcohol as a coping mechanism. The objective of this study was to examine if stress and anxiety were associated with changes in alcohol use in a sample of adult twins. Twins allowed us to control for genetic and shared environmental factors that would confound the alcohol - mental health relationship. Twins (N = 3,971; 909 same-sex pairs) from the Washington State Twin Registry (WSTR) completed an online survey examining several health-related behaviors and outcomes and their self-reported changes due to COVID-19. About 14% of the respondents reported an increase in alcohol use. We found an association between both stress and anxiety and increased alcohol use, where twins with higher levels of stress and anxiety were more likely to report an increase in alcohol consumption. The associations were small and confounded by between-family factors and demographic characteristics. However, there was no significant difference in stress or anxiety levels between non-drinkers and those who reported no change in alcohol use. Our findings suggest that individuals’ mental health may be associated with changes in alcohol use during the COVID-19 pandemic.

## Introduction

The novel coronavirus (COVID-19) has impacted the lives of people worldwide since being declared a pandemic on March 11, 2020 (1). Social restrictions have been put in place to flatten the curve, including the closure of schools, parks, and non-essential businesses^1^. These restrictions may have been successful in slowing the spread of new infections. However, the impact of social isolation and lockdown measures may exacerbate mental health problems such as stress and anxiety, which, in turn, may increase alcohol use as a coping mechanism.

There is an extensive literature on the use of alcohol as a coping mechanism in response to stressful life events at the micro level, such as divorce (2), unemployment (3, 4), and social isolation (5), and at the macro level, such as terrorist attacks (6–9), natural disasters (10–13), and economic recessions (3, 14–16). These studies consistently found an increase in alcohol use, specifically heavy drinking, among individuals exposed to stressful or traumatic events. As alcohol reduces the body’s stress response and emotional memory (17), individuals may consume alcohol to remedy stressful memories related to traumatic events. Longitudinal studies of individuals exposed to a single traumatic event, such as a terrorist attack, found that post-traumatic stress symptoms were associated with an increase in alcohol use over time (7, 9, 13).

Only a handful of studies have investigated the use of alcohol in response to virus outbreak-related stress and anxiety. Among hospital employees in China exposed to the 2003 SARS-CoV outbreak, being quarantined and working in a high-risk location were significantly associated with more alcohol use, with 6% of respondents reporting using alcohol to cope with negative feelings (18). A survey of adults living in Hong Kong during the 2003 SARS-CoV outbreak found that 6.8% of adults reported an increase in alcohol use due to SARS (19).

Regarding the current COVID-19 pandemic, concerns have been raised about the potential risk of increased alcohol consumption due to increased stress (20–22) and social distancing (21). Among US Amazon MTurk workers, those with higher levels of COVID-19–related anxiety were more likely to use drugs and/or alcohol as a coping strategy (23). Among a sample of 4,276 university students in the US surveyed at the end of March 2020, those with more symptoms of depression and anxiety reported a greater increase in alcohol consumption compared to those with fewer symptoms (24). Although increases in alcohol consumption were associated with higher levels of anxiety, depression, and stress symptoms among a sample of 1,491 anonymously surveyed Australian adults in April 2020 (25), a different study among 4,462 Australian adults conducted around the same time found that only depression and stress, but not anxiety, were indicators of a reported increase in alcohol use (26). To date, no studies have examined changes in alcohol use during COVID-19 in a genetically informed sample of adults.

The objective of this study was to examine whether stress and anxiety was associated with perceived changes in alcohol use over the short-term in response to the COVID-19 outbreak and its mitigation strategies in a community-based sample of adult twins primarily residing in the US. We hypothesized that stress and anxiety would be associated with increased alcohol use as a coping strategy. Specifically, we expected that individuals with higher stress and anxiety levels would be more likely to increase the use of alcohol. On the other hand, we expected that those with lower stress and anxiety levels would be more likely to report a decrease in the use of alcohol or report no use of alcohol.

## Methods

### Participants

A total of 3,971 individuals from the Washington State Twin Registry (WSTR) completed an online survey examining several health-related behaviors and outcomes and their self-reported changes due to COVID-19 mitigation, administered between March 26 and April 5, 2020. The survey was sent to 12,173 individuals registered and active in the WSTR; the individual response rate was 32.8% and the pair-wise response rate was 21.2%^2^. The WSTR is a community-based Registry of twin pairs primarily recruited through Washington State Department of Licensing (DOL) records. Details regarding the recruitment procedures of the WSTR and additional information are reported elsewhere (27–29). This study was reviewed and approved by Washington State University Institutional Review Board.

Both monozygotic (MZ, identical) and dizygotic (DZ, fraternal) twins participated in the study. The current sample included 909 same-sex twin pairs (77% MZ, 23% DZ). Zygosity was determined using five questions in the WSTR enrollment survey asking about childhood similarity. Compared to biological zygosity indicators, the survey items correctly classify zygosity with at least 95% accuracy (30, 31).

### Measures

#### Change in Alcohol Use

Participants responded to a series of questions, “Compared to a few weeks ago (i.e., prior to the spread of COVID-19), and thinking only about the past 7 days, please indicate whether you have made changes in the following behaviors.” Several activities and behaviors were assessed. For the current study, we utilized their responses to the “consume alcohol” activity, with four possible response categories: doing more, doing the same, doing less, and do not do.

#### Perceived Stress

We used the 10-item Perceived Stress Scale [PSS; (32)] to assess participants’ stress levels. Participants were asked about their feelings and thoughts in the last 2 weeks with five response categories; 0, never; 1, almost never; 2, sometimes; 3, fairly often; 4, very often. A total PSS score (range = 0 to 40) can be obtained by summing across all scale items, with higher scores indicating higher levels of stress.

#### Anxiety

The six-item anxiety subscale in the Brief Symptom Inventory [BSI; (33)] was used to assess anxiety. Participants were asked to indicate how much discomfort each problem has caused them during the past 2 weeks including today on a five-point Likert-type scale (0 = Not at all; 1 = A little bit; 2 = Moderately; 3 = Quite a bit; 4 = Extremely). A total anxiety score (range = 0 to 24) was computed by summing across all items, where higher scores reflect higher levels of anxiety.

#### Covariates

Participants’ age and sex were included as covariates in the statistical analyses. Age referred to individuals’ age at which they completed the survey; it was computed based on the reported date of birth. Sex was self-reported as male or female.

### Statistical Analysis

In order to examine whether the odds of change in alcohol use is associated with mental health, we performed the following comparisons separately for perceived stress and anxiety: (i) do not use versus use more, (ii) do not use versus use the same, (iii) do not use versus use less, (iv) use the same versus use more, and (v) use the same versus use less.

We first used the classical twin model to decompose the variances of perceived stress, anxiety, and the change in alcohol use into additive genetic (A), shared environmental (C), and non-shared environmental (E) components (34). The A variance components represent the additive effect of genes, with correlation *r* = 1.0 between MZ twins (who share 100% of their genetic sequence) and *r* = 0.5 between DZ twins (who, on average, share 50% of their segregating genes). The C variance components represent common environmental experiences that make members of the same family more similar; they correlate at *r* = 1.0 for both MZ and DZ twins. The E variance components represent non-shared environmental experiences and do not correlate between twins. Measurement error is also included in the E variance components.

**Figure 1:**
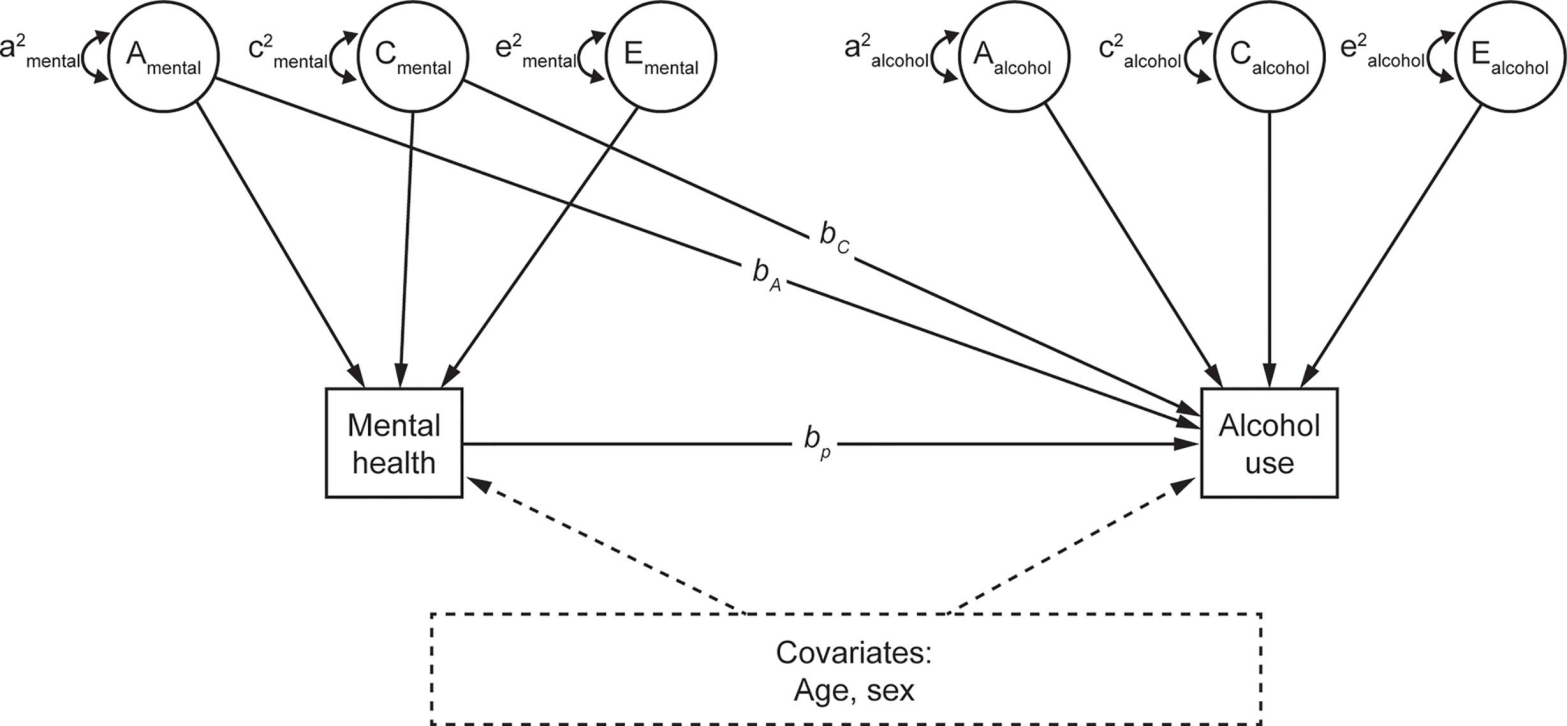
Quasi-causal twin model, controlling for age and sex. A: additive genetic component; C: shared environmental component; E: unique environmental component; *b_A_* and *b_C_*: amount of residual variance of mental health attributable to the genetic and shared environment, respectively; *b_p_*: phenotypic association. Mental health refers to perceived stress or anxiety, in separate models.

We next used phenotypic regression models to examine the association between mental health and change in alcohol use (**Figure 1**). Change in alcohol use was regressed on mental health (i.e., perceived stress or anxiety), estimating the observed association between mental health and change in alcohol use (*b_p_* in **Figure 1**). *b_p_* reflects the phenotypic association between mental health and change in alcohol use, without including genetic or shared environmental confounds.

The models were then re-estimated including estimates of *b_A_* and *b_C_*, respectively controlling for genetic and shared environmental confounds, in the estimation of the phenotypic effect (**Figure 1**). These are referred to as quasi-causal models; the logic and associated statistical methods are described in (35). The *b_A_* and *b_C_* regression paths from perceived stress to change in alcohol use were initially estimated with large standard errors, reflecting a high degree of correlation between the additive genetic (A) and shared environmental (C) components of stress and insufficient power to differentiate between these sources of covariation. *b_A_* and *b_C_* paths from perceived stress to change in alcohol use were subsequently constrained to be the same, meaning that the total between-family effect was estimated instead of individual between-family components. A final set of models were performed by including participants’ age and sex as covariates. Perceived stress and anxiety were both square root transformed as the two variables are positively skewed.

Descriptive statistics were provided for both the full sample and the same-sex twins sample, whereas twin analyses were performed only on the same-sex twins sample. Descriptive statistics were performed in the statistical program R 3.5.3 (36). All latent variable path analyses were conducted using the computer program Mplus v. 8.1 (37). The alpha level for testing hypotheses was set to 0.05. Twin-based regression models are generally saturated; the only source of reduced fit involves incidental issues such as differences between twins arbitrarily assigned as Twin 1 and Twin 2 within pairs. All reported models fit the data closely using standard “goodness of fit” tests.

## Results

### Descriptive Statistics

**Figure 2:**
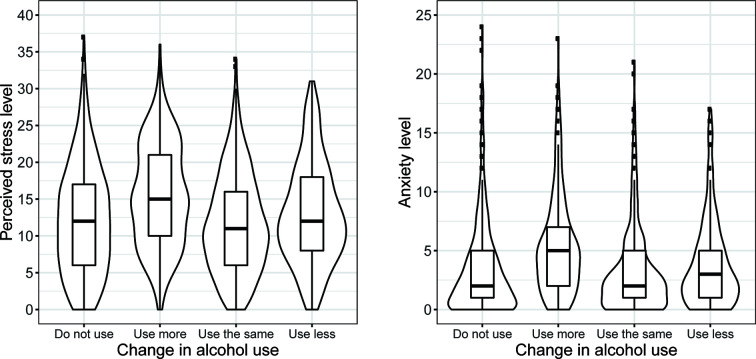
Stress and anxiety levels by self-reported change in alcohol use (same-sex twin pairs).

Descriptive statistics for select demographic characteristics, perceived stress, anxiety, and the proportion of participants with varying changes in alcohol use for the full sample and among same-sex twin pairs are shown in **Table 1**. Most of the participants reported either not using alcohol (35.5% and 36% in full sample and same-sex twins sample, respectively) or using about the same amount (39.4% and 38.3% in full sample and same-sex twins sample, respectively), whereas smaller proportions reported using more (14.3% and 15.3% in full sample and same-sex twins sample, respectively), and even smaller proportions reported using less alcohol (~10% in full sample and same-sex twins sample). The distributions of stress and anxiety levels, by different changes in alcohol use, are presented in **Figure 2**.

**Table 1:** Descriptive statistics of select demographic characteristics, self-report change in alcohol use, perceived stress, and anxiety.

	Full sample (*n* = 3,989)	Same-sex twin pairs (*n* = 909 pairs)
Age	50.4 (*16.0*)	49.9 (*16.0*)
Gender		
Men	1,125 (30.8%)	444 (24.4%)
Women	2,746 (69.2%)	1,374 (75.6%)
White	3,793 (95.5%)	1,738 (95.6%)
Zygosity		
MZ	2,385 (60.1%)	1,400 (77.0%)
DZ	1,586 (39.9%)	418 (23.0%)
Change in alcohol use (%)		
Do not use	1,382 (35.5%)	643 (36.0%)
Use more	556 (14.3%)	274 (15.3%)
Use the same	1,533 (39.4%)	685 (38.3%)
Use less	424 (10.9%)	185 (10.4%)
Perceived stress	12.3 (*7.2*)	12.6 (*7.2*)
Anxiety	3.6 (*3.6*)	3.8 (*4.0*)

Means (standard deviations) are presented for continuous variables. Frequencies (proportions) are presented for categorical variables.

### Univariate Twin Models

Twin correlations for perceived stress and anxiety, as well as tetrachoric twin correlations for the five change in alcohol use comparisons are presented in **Table 2**. The standardized biometric variance components for the variables are also shown; variance component estimates that were negative were subsequently set to zero. There was substantial non-shared environmental variance for perceived stress (61%), whereas the genetic (A: 23%) and shared environmental (C: 16%) variance were much smaller and not significantly different from zero. The univariate decomposition of anxiety showed a combination of genetic (A: 42%) and non-shared environmental (E: 58%) variance. For the three comparisons with the do not use group, the non-shared environmental variance, though small, was significantly different from zero (E: 10%, 29%, and 40% for comparing against use more, use the same, and use less, respectively). On the other hand, the additive genetic and shared environmental variance in these three comparisons were estimated with large standard errors, which may suggest unstable estimates and/or insufficient power. The use the same vs. use more comparison showed a combination of shared (C: 53%) and non-shared environmental (E: 47%) variance. There was substantial non-shared environmental variance (E: 81%) in the use the same vs. use less comparison, with a very small proportion of the variance due to additive genetic variance (A: 19%).

**Table 2:** Twin correlations and standardized variance components for negative emotions, and changes in alcohol use among same-sex twin pairs.

	*r*MZ	*r*DZ	*a^2^*	*c^2^*	*e^2^*
Perceived stress	**.39 (.03)**	**.27 (.06)**	.23 (.13)	.16 (.12)	**.61 (.03)**
Anxiety	**.42 (.03)**	**.21 (.02)**	**.42 (.03)**	–	**.58 (.03)**
Change in alcohol use^a^					
Do not use vs. use more	**.90 (.04)**	**.57 (.16)**	**.65 (.32)**	.25 (.31)	**.10 (.04)**
Do not use vs. use the same	**.71 (.05)**	**.46 (.12)**	.50 (.26)	.21 (.25)	**.29 (.05)**
Do not use vs. use less	**.60 (.10)**	.36 (.28)	.48 (.59)	.12 (.56)	**.40 (.10)**
Use the same vs. use more	**.53 (.07)**	**.53 (.07)**	–	**.53 (.07)**	**.47 (.07)**
Use the same vs. use less	.19 (.13)	.09 (.06)	.19 (.13)	–	**.81 (.13)**

Standard errors are presented within parentheses. rMZ, monozygotic twin correlations; rDZ, dizygotic twin correlations. a^2^, c^2^, and e^2^: standardized biometric variance components obtained from classical twin model decomposing the variance of the phenotype into additive genetic, shared environment, and non-shared environment variance, respectively.

a Tetrachoric correlations are presented here due to the dichotomous nature of the comparisons. Bolded numbers indicate estimates that are statistically significant at p < .05.

### Perceived Stress and Change in Alcohol Use

#### Do Not Use vs. Use More

We found a significant phenotypic association between stress and change in alcohol use (*b_p_* = .314, OR = 1.37, *p* <.001; **Table 3A**). Twins who had higher levels of stress were more likely to report using more alcohol than report not using alcohol. When between-family confounds were controlled in the quasi-causal model, the association was reduced and became non-significant (*b_p_* = .107, OR = 1.11, *p* = .067), suggesting that between-family effects confounded the association between stress and change in alcohol use. Results were similar after further controlling for age and sex (*b_p_* = .116, OR = 1.12, *p* = .062).

**Table 3A:** Unstandardized parameter estimates for phenotypic and biometric models estimating the effects of self-report change in alcohol use on perceived stress.

		Do not use vs. use more			Do not use vs. use the same			Do not use vs. use less		
		Est	OR [95% CI]	*p*	Est	OR [95% CI]	*p*	Est	OR [95% CI]	*p*
Phenotypic model										
	*b_p_*	.314	1.37 [1.24, 1.51]	<.001	−.010	.99 [.93, 1.06]	.762	.076	1.08 [.98, 1.19]	.134
Quasi-causal model										
	*b_A_*	.461	1.59 [1.17, 2.16]	.003	.121	1.13 [.90, 1.42]	.300	−.064	.94 [.67, 1.31]	.704
	*b_C_*	.461	1.59 [1.17, 2.16]	.003	.121	1.13 [.90, 1.42]	.300	−.064	.94 [.67, 1.31]	.704
	*b_p_*	.107	1.11 [.99, 1.25]	.067	−.065	.94 [.86, 1.02]	.141	.106	1.11 [.95, 1.30]	.171
Quasi-causal model (with covariates)										
	*b_A_*	.142	1.15 [.74, 1.80]	.534	.128	1.14 [.82, 1.58]	.443	−.106	.90 [.56, 1.45]	.661
	*b_C_*	.142	1.15 [.74, 1.80]	.534	.128	1.14 [.82, 1.58]	.443	−.106	.90 [.56, 1.45]	.661
	*b_p_*	.116	1.12 [.99, 1.27]	.062	−.066	.94 [.86, 1.02]	.148	.115	1.12 [.96, 1.31]	.149
	Age	−.230	.79 [.73,.86]	<.001	−.050	.95 [.90, 1.0]	.081	.007	1.01 [.93, 1.08]	.855
	Sex (F)	.253	1.29 [.98, 1.68]	.065	−.132	.88 [.73, 1.06]	.166	−.085	.92 [.72, 1.16]	.481
RMSEA [90%CI]		.019 [0,.041]			.017 [0,.040]			.012 [0,.038]		

Phenotypic model does not include controls for between-pair confounds, whereas quasi-causal model include controls for between-pair confounds. Perceived stress is square root transformed; age is divided by 10.

OR, odds ratio; b_A_, amount of variance in perceived stress attributable to additive genetic influences; b_C_, amount of variance in perceived stress attributable to shared environmental influences; b_P_, phenotypic association between predictor and outcome; RMSEA, root mean square error of approximation.

**Figure 3:**
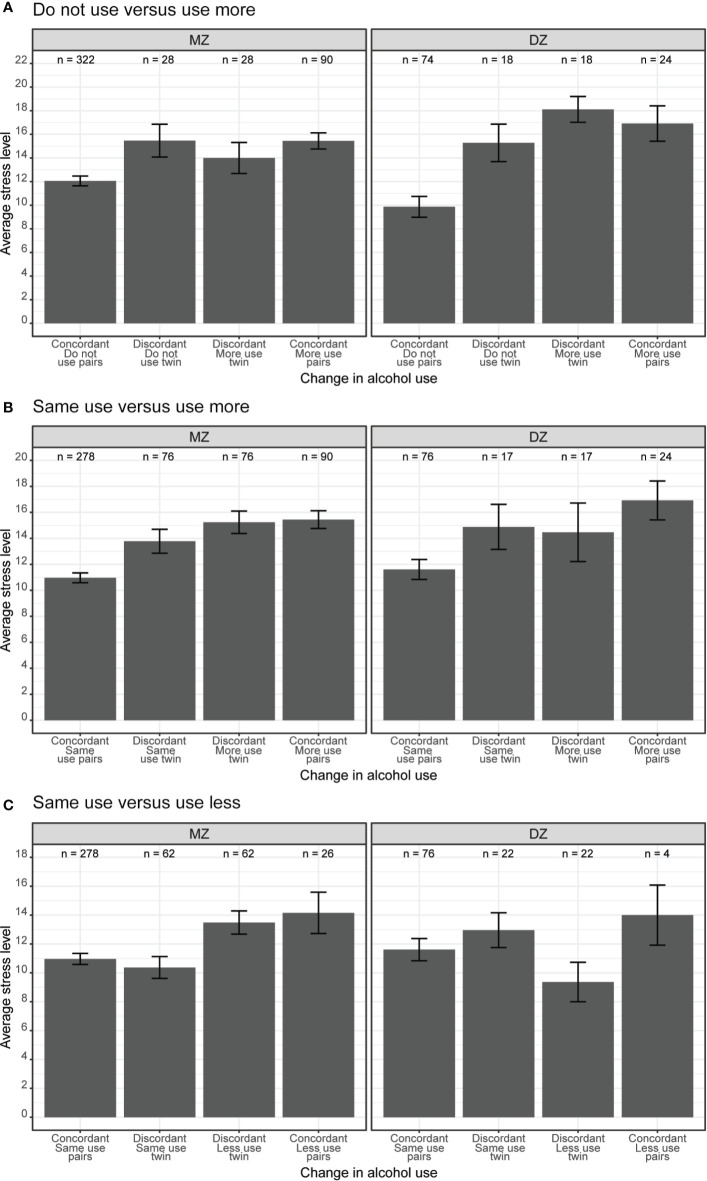
**(A)** Do not use versus use more, **(B)** Same use versus use more, **(C)** Same use versus use less. Average perceived stress levels between twin pairs concordant and discordant in change in alcohol use among same-sex MZ and DZ twin pairs. Error bars denote standard errors.

As shown in **Figure 3A**, there was an overall association between stress and change in alcohol use. Twin pairs where both members reported using more alcohol (rightmost bar in both panels) had higher average stress levels when compared to twin pairs where both members reported not using alcohol (leftmost bar in both panels). However, there was no substantial difference in stress levels among twin pairs discordant in alcohol use (i.e., one member of the pair with increased alcohol use and the other member with no alcohol use, middle two bars in both panels).

#### Do Not Use Versus Use the Same

We found no evidence of an association between stress levels and the odds of not using alcohol versus using the same amount (**Table 3A**). Results were similar in the phenotypic model (*b_p_* = −.010, OR = .99, *p* = .762), the quasi-causal model (*b_p_* = −.065, OR = .94, *p* = .141), and the final model controlling for age and sex (*b_p_* = −.066, OR = .94, *p* = .148).

#### Do Not Use Versus Use Less

There was no association between stress levels and the odds of not using alcohol versus using less alcohol (**Table 3A**). Results were similar in the phenotypic model (*b_p_* = .076, OR = 1.08, *p* = .134), the quasi-causal model (*b_p_* = .106, OR = 1.11, *p* = .171), and the final model controlling for age and sex (*b_p_* = .115, OR = 1.12, *p* = .149).

#### Use the Same Versus Use More

There was a significant phenotypic association between stress and change in alcohol use (*b_p_* = .373, OR = 1.45, *p* <.001; **Table 3B**). Twins with higher levels of stress were more likely to report an increase in alcohol use rather than similar alcohol use. When between-family confounds were controlled in the quasi-causal model, the association was attenuated but remained significant (*b_p_* = .203, OR = 1.23, *p* = .002). Results remained consistent after further controlling for age and sex (*b_p_* = .216, OR = 1.24, *p* = .002).

**Table 3B:** Unstandardized parameter estimates for phenotypic and biometric models estimating the effects of self-report change in alcohol use on perceived stress.

		Use same vs. use more			Use same vs. use less		
		Est	OR [95% CI]	*p*	Est	OR [95% CI]	*p*
Phenotypic model							
	*b_p_*	.373	1.45 [1.32, 1.60]	<.001	.103	1.11 [1.0, 1.22]	.038
Quasi-causal model							
	*b_A_*	.382	1.47 [1.07, 2.00]	.017	−.188	.83 [.59, 1.17]	.288
	*b_C_*	.382	1.47 [1.07, 2.00]	.017	−.188	.83 [.59, 1.17]	.288
	*b_p_*	.203	1.23 [1.08, 1.39]	.002	.191	1.21 [1.03, 1.43]	.022
Quasi-causal model (with covariates)							
	*b_A_*	.078	1.08 [.69, 1.70]	.735	−.244	.78 [.48, 1.28]	.332
	*b_C_*	.078	1.08 [.69, 1.70]	.735	−.244	.78 [.48, 1.28]	.332
	*b_p_*	.216	1.24 [1.08, 1.43]	.002	.202	1.22 [1.03, 1.45]	.019
	Age	−.180	.84 [.77,.90]	<.001	.056	1.06 [.98, 1.14]	.142
	Sex (F)	.369	1.45 [1.13, 1.85]	.003	.025	1.03 [.82, 1.28]	.825
RMSEA [90%CI]		.018 [0,.041]			.025 [0,.045]		

Phenotypic model does not include controls for between-pair confounds, whereas quasi-causal model include controls for between-pair confounds. Perceived stress is square root transformed; age is divided by 10.

OR, odds ratio; b_A_, amount of variance in perceived stress attributable to additive genetic influences; b_C_, amount of variance in perceived stress attributable to shared environmental influences; b_P_, phenotypic association between predictor and outcome; RMSEA, root mean square error of approximation.

We illustrate these associations in **Figure 3B**. Twin pairs who were concordant on more use (i.e., both members reported drinking more; rightmost bar in each panel) had higher average stress levels than concordant same use twin pairs (i.e., both members reported drinking the same amount; leftmost bar in each panel). Among discordant MZ twins (left panel), members of the pair who reported using more alcohol (third bar from the left) had slightly higher stress levels as compared to their co-twins who reported using the same amount of alcohol (second bar from the left). There was no observable difference in stress levels among discordant DZ twins (middle two bars in right panel). As between-pair confounds are controlled within MZ twin pairs, this offers robust evidence for a quasi-causal association between stress levels and change in alcohol use, specifically between same versus increased alcohol use.

#### Use the Same Versus Use Less

We found a significant phenotypic association between stress and change in alcohol use (*b_p_* = .103, OR = 1.11, *p* = .038; **Table 3B**). Twins with higher stress levels were more likely to report a decrease in alcohol use instead of similar alcohol use. This association remained statistically significant after controlling for between-family confounds (*b_p_* = .191, OR = 1.21, *p* = .022), and further controlling for age and sex (*b_p_* = .202, OR = 1.22, *p* = .019).

The phenotypic association between stress levels and change in alcohol use is illustrated in **Figure 3C**. Twin pairs who were concordant on less use (i.e., both members reported drinking less; rightmost bar in each panel) had higher average stress levels than concordant same use twin pairs (i.e., both members reported using same amount of alcohol; leftmost bar in each panel). We observed the same association within pairs of MZ twins discordant for alcohol use – members of the pair who reported drinking less alcohol had substantially higher stress levels than their co-twins who reported drinking the same amount of alcohol (middle two bars in left panel). Within pairs of discordant DZ twins, the average stress levels were higher among members of the pair who reported dinking the same amount of alcohol than their co-twins who reported drinking less alcohol (middle two bars in the right panel). This difference between MZ and DZ discordant twin pairs reflects the genetic confounds, as the between-pair confounds are controlled within discordant MZ twins, and within-pair difference between discordant DZ twins also includes the genetic difference between them.

### Anxiety and Change in Alcohol Use

#### Do Not Use Versus Use More

There was a significant phenotypic association between anxiety and change in alcohol use (*b_p_* = .351, OR = 1.42, *p* <.001; **Table 4A**). Twins with higher levels of anxiety were more likely to report using more alcohol than report not using alcohol. When additive genetics confounds were controlled in the quasi-causal model, the association was reduced and became non-significant (*b_p_* = .119, OR = 1.13, *p* = .135), suggesting that between-family effects confounded the association between stress and change in alcohol use. Results remained similar after further controlling for age and sex (*b_p_* = .139, OR = 1.15, *p* = .086).

**Table 4A:** Unstandardized parameter estimates for phenotypic and biometric models estimating the effects of self-report change in alcohol use on anxiety.

		Do not use vs. use more			Do not use vs. use the same			Do not use vs. use less		
		Est	OR [95% CI]	*p*	Est	OR [95% CI]	*P*	Est	OR [95% CI]	*p*
Phenotypic model										
	*b_p_*	.351	1.42 [1.29, 1.56]	<.001	−.006	.99 [.92, 1.07]	.884	.087	1.09 [.99, 1.20]	.086
Quasi-causal model										
	*b_A_*	.444	1.56 [1.10, 2.21]	.013	.076	1.08 [.85, 1.36]	.521	.090	1.09 [.78, 1.23]	.599
	*b_p_*	.119	1.13 [.96, 1.32]	.135	−.047	.95 [.86, 1.06]	.386	.038	1.04 [.88, 1.23]	.660
Quasi-causal model (with covariates)										
	*b_A_*	.178	1.19 [.77, 1.86]	.430	.092	1.10,.81, 1.48]	.549	.138	1.15 [.76, 1.74]	.518
	*b_p_*	.139	1.15 [.98, 1.35]	.086	−.046	.96 [.86, 1.06]	.388	.038	1.04 [.88, 1.22]	.649
	Age	−.238	.79 [.72,.86]	<.001	−.042	.96 [.91, 1.01]	.131	−.014	.99 [.92, 1.06]	.682
	Sex (F)	.228	1.26 [.95, 1.67]	.114	−.134	.87 [.72, 1.06]	.168	−.070	.93 [.72, 1.21]	.592
RMSEA [90%CI]		.026 [0,.046]			.012 [0,.037]			.015 [0,.039]		

Phenotypic model does not include controls for between-pair confounds, whereas quasi-causal model include controls for between-pair confounds. Perceived stress is square root transformed; age is divided by 10.

OR, odds ratio; b_A_, amount of variance in perceived stress attributable to additive genetic influences; b_C_, amount of variance in perceived stress attributable to shared environmental influences; b_P_, phenotypic association between predictor and outcome; RMSEA, root mean square error of approximation.

**Figure 4:**
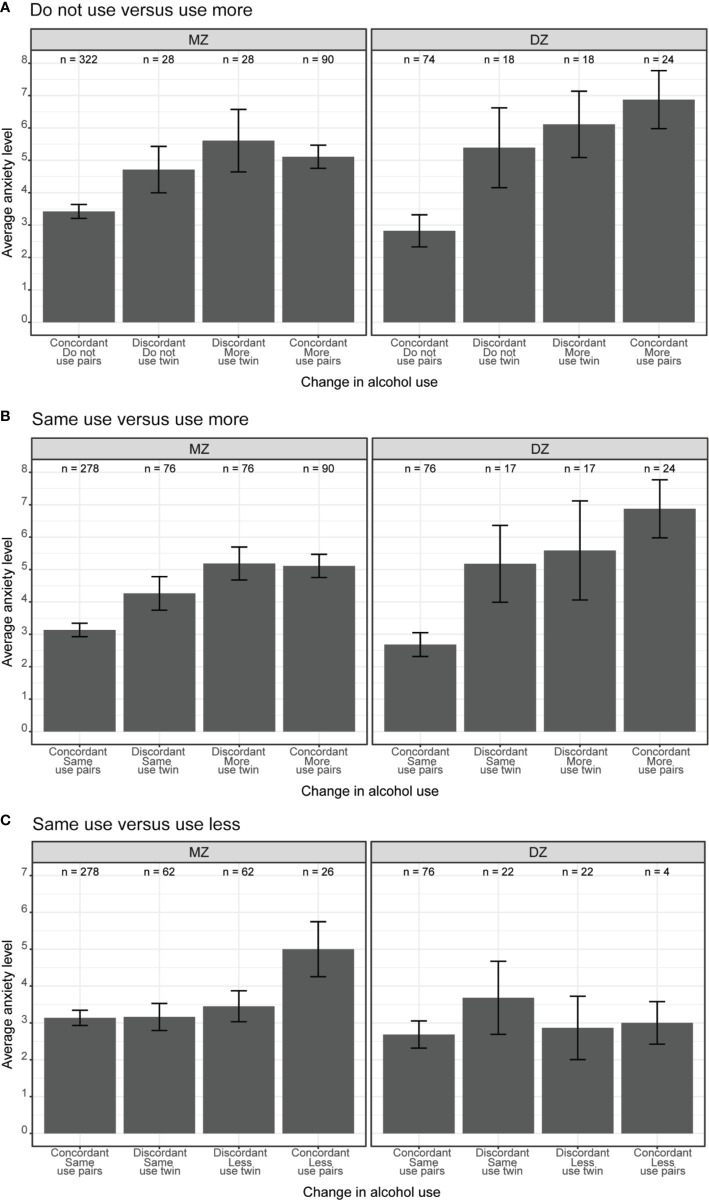
**(A)** Do not use versus use more, **(B)** Same use versus use more, **(C)** Same use versus use less. Average anxiety levels between twin pairs concordant and discordant in change in alcohol use among same-sex MZ and DZ twin pairs. Error bars denote standard errors.

The phenotypic association between anxiety levels and change in alcohol use is illustrated in **Figure 4A**. The average anxiety levels were substantially higher among concordant more use twins (i.e., both members of the pair reported using more alcohol; rightmost bars in both panels) than concordant do not use twins (i.e., both members of the pair reported not drinking; leftmost bars in both panels). However, there was no observable differences in anxiety levels within twin pairs discordant in alcohol use (i.e., one member of the pair with increased use of alcohol and the other member reported not using alcohol, middle two bars in both panels).

#### Do Not Use Versus Use the Same

We found no evidence of an association between anxiety levels and the odds of not using alcohol versus using the same amount (**Table 4A**). Results were similar in the phenotypic model (*b_p_* = −.006, OR = .99, *p* = .884), the quasi-causal model (*b_p_* = −.047, OR = .95, *p* = .386), and the final model controlling for age and sex (*b_p_* = −.046, OR = .96, *p* = .131).

#### Do Not Use Versus Use Less

There was no association between anxiety levels and the odds of not using alcohol versus using less alcohol (**Table 4A**). Results were similar in the phenotypic model (*b_p_* = .087, OR = 1.09, *p* = .086), the quasi-causal model (*b_p_* = .038, OR = 1.04, *p* = .660), and the final model controlling for age and sex (*b_p_* = .038, OR = 1.04, *p* = .649).

#### Use the Same Versus Use More

There was a significant phenotypic association between anxiety and change in alcohol use (*b_p_* = .385, OR = 1.47, *p* <.001; **Table 4B**). Twins with higher levels of anxiety were more likely to report an increase in alcohol use rather than similar alcohol use. When additive genetics confounds were controlled in the quasi-causal model, the association was attenuated and became non-significant (*b_p_* = .147, OR = 1.16, *p* = .080), suggesting that between-family effects confounded the association between anxiety and change in alcohol use. However, we found a significant phenotypic association (*b_p_* = .175, OR = 1.19, *p* = .041) when age and sex were included in the model.

**Table 4B:** Unstandardized parameter estimates for phenotypic and biometric models estimating the effects of self-report change in alcohol use on anxiety.

		Use same vs. use more			Use same vs. use less		
		Est	OR [95% CI]	*p*	Est	OR [95% CI]	*p*
Phenotypic model							
	*b_p_*	.385	1.47 [1.34, 1.61]	<.001	.098	1.10 [1.00, 1.21]	.045
Quasi-causal model							
	*b_A_*	.457	1.58 [1.11, 2.26]	.012	.047	1.05 [.74, 1.48]	.790
	*b_p_*	.147	1.16 [.98, 1.37]	.080	.073	1.08 [.90, 1.28]	.412
Quasi-causal model (with covariates)							
	*b_A_*	.173	1.19 [.76, 1.85]	.441	.087	1.09 [.71, 1.67]	.685
	*b_p_*	.175	1.19 [1.01, 1.41]	.041	.073	1.08 [.91, 1.28]	.408
	Age	−.207	.81 [.75,.88]	<.001	.019	1.02 [.95, 1.09]	.575
	Sex (F)	.364	1.44 [1.11, 1.87]	.006	.054	1.08 [.91, 1.28]	.654
RMSEA [90%CI]		.027 [0,.047]			.022 [0,.043]		

Phenotypic model does not include controls for between-pair confounds, whereas quasi-causal model include controls for between-pair confounds. Perceived stress is square root transformed; age is divided by 10.

OR, odds ratio; b_A_, amount of variance in perceived stress attributable to additive genetic influences; b_C_, amount of variance in perceived stress attributable to shared environmental influences; b_P_, phenotypic association between predictor and outcome; RMSEA, root mean square error of approximation.

The main effect of anxiety on change in alcohol use is shown in **Figure 4B**; the average anxiety levels were higher among concordant more use twin pairs (i.e., both members of the pair reported using more alcohol; rightmost bar in both panels) than concordant same use twin pairs (i.e., both members of the pair reported using same amount of alcohol; leftmost bar in both panels). When comparing twin pairs discordant in alcohol use (i.e., one member of the pair using more alcohol, and their co-twin using same amount of alcohol), there was no substantial differences in anxiety levels (middle bars in both panels).

#### Use the Same Versus Use Less

There was a small phenotypic association between anxiety and change in alcohol use (*b_p_* = .098, OR = 1.10, *p* = .045; **Table 4B**). Twins with higher stress levels were more likely to report a decrease in alcohol use instead of similar alcohol use. This association was reduced and became non-significant after controlling for additive genetics confounds (*b_p_* = .073, OR = 1.08, *p* = .412), and age and sex (*b_p_* = .073, OR = 1.08, *p* = .408).

As shown in **Figure 4C**, MZ twin pairs where both members reported using less alcohol (rightmost bar in left panel) had higher levels of anxiety, compared to MZ twins where both members reported using the same amount of alcohol (leftmost bar in left panel). This association was reduced among concordant DZ twin pairs (right panel). The average anxiety levels were similar among discordant twin pairs, regardless of alcohol use (middle two bars in both panels).

## Discussion

Results of this study showed a significant association between stress and anxiety levels and increased alcohol use. Twins with higher levels of stress and anxiety were more likely to report an increase in alcohol consumption, instead of no alcohol consumption. These phenotypic associations were no longer significant after controlling for between-family confounds, suggesting that the associations were mediated by between-family factors. Stress and anxiety levels did not have a substantial impact on whether twins report no versus similar amount of alcohol consumption, or no versus reduced alcohol consumption.

Among twins who drink, higher levels of stress were associated with higher odds of increased versus same alcohol use. This association was robust after controlling for between-family effects: members of the pair with higher levels of stress were more likely to drink more than their co-twins with lower levels of stress. Contrary to our expectation, twins with higher levels of stress were also more likely to report decreased, rather than same, alcohol consumption. This phenotypic association remained significant after controlling for between-family confounds, meaning that members of the pair with higher levels of stress were more likely to drink less than their co-twins with lower stress levels. Similar associations were observed between anxiety levels and change in alcohol use, though the relationships were confounded by between-family effects.

Our study showed that 14.3% of the respondents reported an increase in alcohol consumption, which is comparable with existing studies that reported an increase in alcohol use among individuals exposed to the SARS outbreak (6, 7). These two studies further showed that stress related to the outbreak was linked to increased alcohol consumption. Although the cross-sectional nature of the data in this study precludes us from drawing conclusions regarding the direction of the association, we also showed that stress and anxiety levels are linked to increased alcohol consumption. The current study further showed that stress and anxiety levels associated with the COVID-19 outbreak may have an acute impact on individuals—an increase in alcohol consumption was reported only 2 weeks after the WHO declared the COVID-19 outbreak a pandemic (1). Although alcohol use may be an effective coping strategy in the short term (38), persistent increased alcohol consumption may turn into problematic behaviors, such as alcohol dependence and/or abuse. With prior studies showing increased alcohol use shortly after (7), and up to three years (6) after the SARS outbreak, it would be important to investigate the extent to which the current COVID-19 pandemic may be associated with increased alcohol use in the long term. Considering that almost every country in the world has been affected by the COVID-19 outbreak, it is essential that strategies are put in place to prevent problematic alcohol use behaviors. Longitudinal studies would provide additional information about the changes in alcohol consumption as the world recovers from the pandemic, and determine if specific personality and/or health factors are associated with whether individuals return to their normal amount of consumption or continue to be dependent on alcohol.

### Strengths and Limitations

The timeliness of the survey is one of the biggest strengths of the current study. The survey was administered during a 2-week period in late March and early April 2020, less than a month after the COVID-19 was declared a pandemic by the World Health Organization (1). We were able to assess the immediate impact of the COVID-19 pandemic and the corresponding social restrictions on stress and anxiety levels, as well as changes in alcohol use in a relatively large sample of adult twins.

The current study asked participants to report their perceived change in alcohol use, providing a subjective assessment of the extent to which alcohol use has changed or remained the same. Although the subjective assessment may suffer from response bias (e.g., individuals may be reluctant to report increased use of alcohol), slightly more participants reported an increase in alcohol use (~15%) than a decrease in alcohol use (~10%), suggesting that twins in our sample may not necessarily be reluctant to report an increase in alcohol use. As it is not possible to accurately assess participants’ alcohol use prior to the COVID-19 pandemic, individuals’ perceived change in alcohol use may reasonably reflect their actual changes in alcohol use. Additionally, the WSTR is planning to conduct follow-up studies to examine how individuals’ mental health and everyday behaviors change in response to the ease of social restriction measures. When data from the longitudinal studies becomes available, we will be able to investigate the extent to which alcohol use changes over time, and whether perceived change in alcohol use corresponds to individuals’ actual change in alcohol use during this time.

We recognize that the current study may potentially suffer from self-selection bias. Although the response rate for this study was comparable to prior WSTR studies, only about one-third of the individuals registered in the WSTR completed the survey. It is possible that individuals who responded to our survey invitation were less stressed and/or anxious, as reflected by the relatively low stress (M = 12.6 out of a maximum of 40) and anxiety (M = 3.8 out of a maximum of 24) levels in the current study. We examined survey results of 2,000 individuals who completed a prior WSTR survey within one year of this study. There was no statistically significant difference in alcohol use as measured by the Alcohol Use Disorders Identification Test (AUDIT-C) (39) (b = −.01, SE = .04, p = .715), perceived stress (b = .11, SE = .06, p = .063), or anxiety (b = −.02, SE = .05, p = .641) between individuals who participated in the current study (N = 1,384)^3^ and those who did not (N = 616). These results suggest that participants in the current study may not be a particularly low stress/anxiety or low alcohol consumption group of individuals prior to the pandemic, as compared to those who did not participate in this study. Nonetheless, with no current available information on non-responders, we are unable to speculate whether individuals who did not participate in the current study had higher (or lower) levels of stress and anxiety, and whether their alcohol consumption had changed or remained unchanged during this time period. We are also unable to determine whether similar associations between mental health and alcohol use would be replicated among other samples with higher levels of stress and anxiety, or samples from other populations.

## Conclusion

The current study investigated the extent to which individuals’ stress and anxiety levels were associated with self-reported change in the amount of alcohol use. We found that twin pairs with higher levels of stress and anxiety were more likely to report an increase in alcohol use rather than no alcohol use or a similar amount of alcohol use. Those with higher stress and anxiety levels were also more likely to report a decrease in alcohol use instead of a similar amount of alcohol use. Most of these associations were small and confounded by between-family factors (genetic and shared environment factors) and demographic characteristics, such as age and sex. Results from the current study suggest that individuals’ mental health may be associated with changes in alcohol use during this stressful time as people navigate through the COVID-19 pandemic.

## Data Availability Statement

The datasets presented in this article are not readily available because: data is provided by the Washington State Twin Registry after acceptance of a manuscript proposal with a signed data access agreement. Requests to access the datasets should be directed to www.wstwinregistry.org.

## Ethics Statement

The studies involving human participants were reviewed and approved by Washington State University Institutional Review Board. The ethics committee waived the requirement of written informed consent for participation.

## Author Contributions

All authors contributed to the article and approved the submitted version.

## Funding

This work was supported by a grant from the National Institute of Health (grant number R33ES024715).

## Conflict of Interest

The authors declare that the research was conducted in the absence of any commercial or financial relationships that could be construed as a potential conflict of interest.

## Footnotes

1 The definition of essential business and/or service differs across countries and local governments.

2 Response rates in the current study were comparable to prior WSTR survey-based studies (~32% and 21% individual and pair-wise response rate, respectively, across 13 unique studies). Demographic characteristics of the current respondents were like those in the full WSTR (data available upon request).

3 Some individuals who participated in the current study did not complete another survey within the past year.

## References

[1] World Health Organization WHO Director-General’s opening remarks at the media briefing on COVID-19 - 11 March 2020. (2020). Retrieved from https://www.who.int/dg/speeches/detail/who-director-general-s-opening-remarks-at-the-media-briefing-on-covid-19---29-june-2020.

[2] Leonard KE Rothbard JC Alcohol and the marriage effect. J Stud Alcohol (1999) 13:139–46. 10.15288/jsas.1999.s13.139 10225498

[3] Mulia N Zemore SE Murphy R Liu H Catalano R Economic Loss and Alcohol Consumption and Problems During the 2008 to 2009 U.S. Recession. Alcohol: Clin Exp Res (2014) 38(4):1026–34. 10.1111/acer.12301 PMC4018214 24256500

[4] Rosenthal L Carroll-Scott A Earnshaw VA Santilli A Ickovics JR The importance of full-time work for urban adults’ mental and physical health. Soc Sci Med (2012) 75(9):1692–6. 10.1016/j.socscimed.2012.07.003 PMC3504362 22858166

[5] Tanskanen J Anttila T A Prospective Study of Social Isolation, Loneliness, and Mortality in Finland. Am J Public Health (2016) 106(11):2042–8. 10.2105/AJPH.2016.303431 PMC5055788 27631736

[6] Hasin DS Keyes KM Hatzenbuehler ML Aharonovich EA Alderson D Alcohol Consumption and Posttraumatic Stress After Exposure to Terrorism: Effects of Proximity, Loss, and Psychiatric History. Am J Public Health (2007) 97(12):2268–75. 10.2105/AJPH.2006.100057 PMC2089108 17971553

[7] Boscarino JA Kirchner HL Hoffman SN Sartorius J Adams RE PTSD and alcohol use after the World Trade Center attacks: A longitudinal study. J Traumatic Stress (2011) 24(5):515–25. 10.1002/jts.20673 PMC3557517 21882246

[8] Pfefferbaum B E Doughty DE Increased Alcohol Use in a Treatment Sample of Oklahoma City Bombing Victims. Psychiatry: Interpersonal Biol Processes (2001) 64(4):296–303. 10.1521/psyc.64.4.296.18598 11822207

[9] Richman JA Cloninger L Rospenda KM Macrolevel Stressors, Terrorism, and Mental Health Outcomes: Broadening the Stress Paradigm. Am J Public Health (2008) 98(2):323–9. 10.2105/AJPH.2007.113118 PMC2376880 18172139

[10] Flory K Hankin BL Kloos B Cheely C Turecki G Alcohol and Cigarette Use and Misuse Among Hurricane Katrina Survivors: Psychosocial Risk and Protective Factors. Subst Use Misuse (2009) 44(12):1711–24. 10.3109/10826080902962128 PMC2782914 19895302

[11] Cerdá M Tracy M Galea S A prospective population based study of changes in alcohol use and binge drinking after a mass traumatic event. Drug Alcohol Dependence (2011) 115(1-2):1–8. 10.1016/j.drugalcdep.2010.09.011 20977977 PMC3039709

[12] Lowe SR Sampson L Young MN Galea S Alcohol and Nonmedical Prescription Drug Use to Cope With Posttraumatic Stress Disorder Symptoms: An Analysis of Hurricane Sandy Survivors. Subst Use Misuse (2017) 52(10):1348–56. 10.1080/10826084.2017.1280832 28394737

[13] Kanehara A Ando S Araki T Usami S Kuwabara H Kano Y Trends in psychological distress and alcoholism after The Great East Japan Earthquake of 2011. SSM - Popul Health (2016) 2:807–12. 10.1016/j.ssmph.2016.10.010 PMC5757822 29349191

[14] Margerison-Zilko C Goldman-Mellor S Falconi A Downing J Health Impacts of the Great Recession: a Critical Review. Curr Epidemiol Rep (2016) 3(1):81–91. 10.1007/s40471-016-0068-6 27239427 PMC4880023

[15] Dee TS Alcohol abuse and economic conditions: Evidence from repeated cross-sections of individual-level data. Health Econ (2001) 10(3):257–70. 10.1002/hec.588 11288191

[16] Vijayasiri G Richman JA Rospenda KM The Great Recession, somatic symptomatology and alcohol use and abuse. Addictive Behav (2012) 37(9):1019–24. 10.1016/j.addbeh.2012.04.007 PMC3383405 22632797

[17] Stewart SH Mitchell TL Wright KD Loba P The relations of PTSD symptoms to alcohol use and coping drinking in volunteers who responded to the Swissair Flight 111 airline disaster. J Anxiety Disord (2004) 18(1):51–68. 10.1016/j.janxdis.2003.07.006 14725868

[18] Wu P Liu X Fang Y Fan B Fuller CJ Guan Z Alcohol abuse/dependence symptoms among hospital employees exposed to a SARS outbreak. Alcohol Alcohol (2008) 43(6):706–12. 10.1093/alcalc/agn073 PMC2720767 18790829

[19] Lau JT Yang X Pang E Tsui HY Wong E Wing YK SARS-related perceptions in Hong Kong. Emerg Infect Dis (2005) 11(3):417–24. 10.3201/eid1103.040675 PMC3298267 15757557

[20] Rehm J Kilian C Ferreira-Borges C Jernigan D Monteiro M Parry CDH Alcohol use in times of the COVID 19: Implications for monitoring and policy. Drug Alcohol Rev (2020) 39(4):301–4. 10.1111/dar.13074 PMC7267161 32358884

[21] Da BL Im GY Schiano TD COVID-19 Hangover: A Rising Tide of Alcohol Use Disorder and Alcohol-Associated Liver Disease. Hepatology (2020). 10.1002/hep.31307 32369624

[22] Clay JM Parker MO Alcohol use and misuse during the COVID-19 pandemic: a potential public health crisis? Lancet Public Health (2020) 5(5):e259. 10.1016/S2468-2667(20)30088-8 32277874 PMC7195126

[23] Lee SA Mathis AA Jobe MC Pappalardo EA Clinically significant fear and anxiety of COVID-19: A psychometric examination of the Coronavirus Anxiety Scale. Psychiatry Res (2020) 290:113112. 10.1016/j.psychres.2020.113112 32460185 PMC7237368

[24] Lechner WV Laurene KR Patel S Anderson M Grega C Kenne DR Changes in alcohol use as a function of psychological distress and social support following COVID-19 related University closings. Addictive Behav (2020) 110:106527. 10.1016/j.addbeh.2020.106527 PMC7319610 32679435

[25] Stanton R To QG Khalesi S Williams SL Alley SJ Thwaite TL Depression, Anxiety and Stress during COVID-19: Associations with Changes in Physical Activity, Sleep, Tobacco and Alcohol Use in Australian Adults. Int J Environ Res Public Health (2020) 17(11):4065. 10.3390/ijerph17114065 PMC7312903 32517294

[26] Neill E Meyer D Toh WL Rheenen TE Phillipou A Tan EJ Alcohol Use in Australia during the Early Days of the COVID -19 Pandemic: Initial results from the COLLATE project. Psychiatry Clin Neurosci (2020). 10.1111/pcn.13099 PMC7436134 32602150

[27] Duncan GE Avery AR Strachan E Turkheimer E Tsang S The Washington State Twin Registry: 2019 Update. Twin Res Hum Genet (2019) 22(6):788–93. 10.1017/thg.2019.36 31358074

[28] Strachan E Hunt C Afari N Duncan G Noonan C Schur E University of washington twin registry: poised for the next generation of twin research. Twin Res Hum Genet (2013) 16(1):455–62. 10.1017/thg.2012.124 PMC4505360 23218177

[29] Afari N Noonan C Goldberg J Edwards K Gadepalli K Osterman B University of Washington Twin Registry: construction and characteristics of a community-based twin registry. Twin Res Hum Genet (2006) 9(6):1023–9. 10.1375/twin.9.6.1023 PMC2953369 17254446

[30] Eisen S Neuman R Goldberg J Rice J True W Determining zygosity in the Vietnam Era Twin Registry: an approach using questionnaires. Clin Genet (1989) 35(6):423–32. 10.1111/j.1399-0004.1989.tb02967.x 2736790

[31] Torgersen S The determination of twin zygosity by means of a mailed questionnaire. Acta Genet Med Gemellol (Roma) (1979) 28(3):225–36. 10.1017/S0001566000009077 297423

[32] Cohen S Kamarck T Mermelstein R A global measure of perceived stress. J Health Soc Behav (1983) 24(4):385–96. 10.2307/2136404 6668417

[33] Derogatis LR Melisaratos N The Brief Symptom Inventory: an introductory report. Psychol Med (1983) 13(3):595–605. 10.1017/S0033291700048017 6622612

[34] Neale M Cardon L Methodology for Genetic Studies of Twins and Families. Dordrecht N, editor. The Netherlands: Kluwer Academic (1992).

[35] Turkheimer E Harden KP Behavior genetic research methods: Testing quasi-causal hypotheses using multivariate twin data. In: HT R, editor. Handbook of research methods in social and personality psychology, 2nd ed. Cambridge, U.K: Cambridge University Press (2014). p. 159–87.

[36] R Core Team R: A language and environment for statistical computing. 3.5.1 ed. R Foundation for Statistical Computing: Vienna, Austria (2013).

[37] Mutheén L Mutheén B Mplus. Statistical analysis with latent variables. User"s Guide. Muthen & Muthen: Los Angeles, CA (2012).

[38] McFarlane AC Epidemiological evidence about the relationship between PTSD and alcohol abuse: the nature of the association. Addict Behav (1998) 23(6):813–25. 10.1016/S0306-4603(98)00098-7 9801718

[39] Bush K Kivlahan DR McDonell MB Fihn SD Bradley KA The AUDIT Alcohol Consumption Questions (AUDIT-C) An Effective Brief Screening Test for Problem Drinking. Arch Internal Med (1998) 158(16):1789–95. 10.1001/archinte.158.16.1789 9738608

